# The silent crisis: investigating the impact of environmental pollutants on embryo-fetal development: a narrative review of the Group of Special Interest for Environment of the Italian Society of Fertility and Sterility and Reproductive Medicine

**DOI:** 10.1007/s10815-025-03653-9

**Published:** 2025-10-01

**Authors:** Alessandra Alteri, Stefano Canosa, Andrea Di Nisio, Carlo Foresta, Valerio Pisaturo

**Affiliations:** 1 Obstetrics and Gynaecology Unit, IRCCS San Raffaele Scientific Institute, Milan, Italy; 2 IVIRMA, Global Research Alliance, Livet, Turin, Italy; 3 Department of Psychology and Health Sciences, Pegaso University, Naples, Italy; 4 Department of Medicine – DIMED, University of Padova, Via Giustiniani, 2, 35128 Padua, Italy; 5 Department of Maternal and Child Health and Urological Sciences, Sapienza - University of Rome, Rome, Italy

**Keywords:** Endocrine disruptors, Embryo development, Fetus, Pollution, Stress

## Abstract

**Purpose:**

Environmental pollution is a growing global concern, yet its effects on reproductive health remain poorly understood. While numerous epidemiological studies have identified strong associations between exposure to pollutants and adverse reproductive outcomes, the precise biological mechanisms underlying these effects remain elusive.

**Methods:**

A comprehensive literature search was performed by two reviewers using the US National Library of Medicine (NCBI Pubmed) up to August 15, 2025.

**Results:**

Chemical and physical contaminants, including endocrine-disrupting chemicals (EDCs), particulate matter (PM), heavy metals, and heat stress (HS), have been implicated in disrupting of essential reproductive processes, such as gametogenesis, fertilization, implantation, and embryogenesis. Despite accumulating evidence, the complexity of these interactions has hindered the development of targeted interventions and effective regulatory policies.

**Conclusions:**

This review argues that investment in reproductive environmental research is not only necessary but urgent. The pervasive nature of pollution and its potential long-term consequences on fertility and pregnancy outcomes warrant an intensified focus on mechanistic studies, improved environmental monitoring, and the integration of toxicological data into reproductive medicine. It is imperative to prioritize research that can provide the necessary insights to mitigate these threats. This paper highlights the knowledge gaps, emphasizing the need for a multidisciplinary approach that combines epidemiology, toxicology, and molecular biology to inform policies and safeguard reproductive health.

## Introduction

Reproductive health is fundamental to human sustainability, yet it faces an ever-growing threat from environmental pollution. The human reproductive system, particularly during early embryonic development, is susceptible to external insults. Mounting evidence suggests that exposure to environmental pollutants can profoundly affect fertility and pregnancy outcomes [1–3]. Despite decades of research, the precise mechanisms through which pollutants interfere with reproduction remain unclear, leaving significant gaps in our understanding of their long-term consequences [4]. Chemical and physical pollutants, including endocrine-disrupting chemicals (EDCs), particulate matter (PM), heavy metals, and heat stress, have been linked to impaired gametogenesis, altered implantation, and disrupted embryonic development. Epidemiological studies have shown that maternal exposure to these pollutants is associated with increased risks of miscarriage, fetal malformations, and long-term health impairments in offspring [5, 6]. The urgency of this issue extends beyond individual reproductive success and represents a public health crisis with far-reaching implications for population sustainability. Despite clear indications that pollution negatively affects reproductive outcomes, scientific funding and regulatory action remain insufficient. This paper of the Italian Society of Fertility and Sterility and Reproductive Medicine (SIFES-MR) argues that the time to act is now: research on environmental pollutants and reproductive health must be prioritized to develop evidence-based policies and mitigation strategies before it is too late.

## Methods

This is a narrative review presented on behalf of the SIFES-MR by a Group of Interest (GIS) of experts in the field. The writing group includes Italian reproductive physicians working in the field of gynecology, andrology, and endocrinology; embryologists; and scientists with expertise in IVF. A comprehensive literature search was performed by two reviewers in the US National Library of Medicine (NCBI Pubmed) up to August 15, 2025. As for the search terms, including MeSH® terms and MeSH Supplementary Concepts (https://www.nlm.nih.gov/mesh), they comprised acronyms and full names. The terms heavy metals, air pollution (PM 10 or PM 2.5), temperature (heat, global warming, climate), and endocrine disruptors (PFAS or perfluoroalkyl and polyfluoroalkyl substances, PFOA or perfluorooctanoic acid, phthalates, BPA or bisphenol A, bisphenol S and F) were combined with search terms for embryo development and fetal development. Full text publications published in the past 10 years, from 2014, were selected. Pre-clinical and basic research studies were included by screening the abstracts or titles. Publications without abstract, not English written, and duplicates were also removed.

## Endocrine disrupting chemicals and embryo-fetal development

Recent scientific evidence emphasizes that exposure to toxic environmental agents prior to conception and during pregnancy can have profound and lasting effects on an individual’s health and susceptibility to disease in later life [7]. Woodruff et al. [8] highlighted key windows of susceptibility that extend from the periconceptional period, encompassing the time before, during and immediately after fertilization, through various stages of development, including pregnancy, infancy, childhood, and puberty. In this context, the placenta plays a crucial role as a barrier between the mother and the fetus, controlling nutrient delivery, waste removal, and protecting the fetus from harmful substances [9, 10]. Indeed, many environmental pollutants can cross the placental barrier and accumulate in both the placenta and the fetus, which can result in fetal exposure levels exceeding those of the mother [11]. Endocrine-disrupting chemicals (EDCs), which include substances such as phenols, phthalates, parabens, flame retardants, and heavy metals, are exogenous chemicals that have the potential to disrupt hormone production and function, with several studies focusing on the prenatal effects of such exposure [12, 13]. Epidemiological data suggested that exposure to EDCs is almost universal among pregnant women, mainly through the use of personal care products, cosmetics, detergents, electronic devices, and contaminated food [14]. EDCs, including bisphenols, phthalates, perfluoroalkyl substances (PFAS), and heavy metals, have been detected in placental tissue, cord blood, and amniotic fluid, confirming direct fetal exposure. These substances accumulate in maternal and fetal tissues, leading to oxidative stress, inflammation, and epigenetic changes that may have lifelong consequences [15].

### Perfluoroalkyl and polyfluoroalkyl substances

New persistent organic pollutants (POPs) have emerged in which hydrogen atoms are partially or completely replaced by fluorine atoms in straight or branched chains. Due to their distinctive properties, including hydrophobicity, oleophobicity, exceptional chemical, thermal stability, and high surface activity, PFASs have found widespread use in many industries and commercial products, including textiles, papermaking, packaging, pesticides, firefighting foams, household goods, and cosmetics. PFASs are characterized by strong carbon–fluorine bonds, making them highly resistant to degradation and capable of accumulating in organisms through food chains. Numerous studies have shown that PFASs accumulate in protein-rich organs, tissues, and body fluids such as the liver, kidneys, human serum, and breast milk. In addition, PFASs can cross the placental barrier and enter the fetus via umbilical cord blood. Studies indicated that the transfer of PFASs across the placenta during pregnancy may be associated with adverse health effects and birth outcomes for the fetus [16, 17]. Mechanisms such as passive diffusion and active transport have been proposed to explain the transplacental transfer of PFAS [18]. Several studies have suggested that PFASs may adversely affect the placenta through mechanisms such as impaired vasculogenesis and angiogenesis, oxidative stress, imbalance in pro- and anti-apoptotic signaling, impaired trophoblast function, and epigenetic alterations. Impaired placental development or function has been associated with several adverse pregnancy outcomes, including hypertensive disorders, gestational diabetes, and low birth weight [19, 20]. Interestingly, paternal PFAS exposures have also been associated with lower offspring birth weight and shorter birth length [21]. Conversely, there is limited information on the effects of PFASs during the period between fertilization and late pre-implantation embryo development. Progesterone (P4) affects the endometrial lining by promoting its final differentiation and secretory functions, while P4 antagonists affect fertility by inhibiting ovulation and directly affecting the endometrium. This is part of the preparation for implantation during the luteal phase. PFAS, particularly PFOA, can disrupt ovarian function by interfering with P4 production. In particular, Di Nisio et al. [22] showed that PFOA directly interferes with P4 signaling in human endometrial cells, antagonizing progesterone-responsive genes involved in endometrial receptivity and embryo implantation. PFOA exposure has also been shown to inhibit the expression of estrogen sulfotransferase genes, with PFOA antagonizing mRNA levels, which in turn impairs embryo attachment [23].

### Heavy metals

Studies have reported the presence of lead, cadmium, arsenic, and mercury in the placenta, cord blood, and maternal blood of pregnant populations worldwide, regardless of age and time. The accumulation of metals in food, air, and water poses a risk of exposure in humans [24]. The placenta acts as a “gatekeeper” between maternal and fetal exposures [25]. These metals can cross the placental barrier with relative ease, posing significant risks to the developing fetus [26, 27]. These metals can induce oxidative stress, disrupt cell membrane potential, and disturb cellular homeostasis. In fact, they can act as catalysts in redox reactions and are consequently involved in unregulated production of free radicals, such as reactive oxygen species (ROS) and reactive nitrogen species (RNS) in endothelial cells. These free radicals, in turn, damage DNA, membrane lipids, and enzymes in placental tissues. Valko et al. [28] suggested that free radical formation is linked to metal toxicity, which then diminishes the functioning of the defense mechanism. In the formation of placental ROS, aerobic respiration and mitochondrial electron transport metabolism play an important role [29]. In this context, the mechanism underlying the formation of free radicals by toxic metals in the human placental tissue is similar to that in other tissues [30, 31]. Toxic metals exhibit a greater affinity for enzymes, leading to either enzyme inhibition or activation, and are thus responsible for lipid peroxidation in biological tissues [32]. Exposure to high levels of metals like arsenic (As), cadmium (Cd), and lead (Pb) has been strongly associated with adverse pregnancy outcomes, such as an increased risk of low birth weight, which can negatively impact long-term health [33–36]. In addition to these concerns, certain metals, particularly As, Cd, mercury (Hg), and Pb, are recognized neurotoxicants. Prenatal exposure to these metals has been linked to significant impairments in neurodevelopment and cognitive abilities, including delayed learning and memory deficits, which may have lifelong consequences [37–40]. In addition, several studies suggested that such exposure is associated with alterations in DNA methylation in umbilical cord blood at multiple loci. These epigenetic changes have been related to both immediate and long-term health effects in children, including cardiovascular disease (CVD) and neurodevelopmental disorders [41, 42].

Lead (Pb) exposure can occur through household paints, toys, and groundwater contaminated by Pb-containing plumbing. Several indirect mechanisms have been proposed for Pb-induced oxidative stress in the placenta. Pb is similar to a non-redox-reactive toxic metal, that is, this metal cannot directly participate in free radical formation. For instance, it promotes oxidative imbalance through the inhibition of delta-aminolevulinic acid dehydratase, a key enzyme in heme synthesis, and through the reduction of antioxidant activity. Thus, Pb toxicity causes oxidative stress in the placental tissue, leading to placental tissue injury and preterm birth [43, 44].

Cadmium (Cd) can be ingested through drinking water contaminated by soil pollution, industrial activity, waste combustion, or smoking. Cd is responsible for spontaneous miscarriage due to an increase in inflammation, interference with the action of endogenous hormones, and alteration in placental functions, but also plays a key role in reducing blood flow and transfer of nutrition in the placental tissue, thus disturbing the normal growth of the fetus and pregnancy outcome [45]. However, the exact mechanism of Cd-induced cytotoxicity in the placental tissue is not well understood. Furthermore, Cd cannot directly provoke free radicals as it is not a redox-reactive metal. Iron and copper from various cytoplasmic and membrane proteins are disarticulated by Cd, which can increase the amount of liberated, free chelated copper, and iron ions, rendering oxidative stress through Fenton reactions [46, 47]. Elevated levels of placental Cd may induce oxidative stress, leading to injury in vascular endothelial cells of the placenta and consequently resulting in preterm deliveries [48]. Cadmium has been proposed to be one of the potential factors of neurobehavioral alterations [49] and CVDs development [50]. Maternal cadmium exposure during critical windows of development has been associated with long-term alterations that increase the risk of hypertension and CVDs in adulthood [50]. A recent human cohort study showed that higher maternal blood cadmium levels during pregnancy are associated with DNA methylation changes in cord blood at genes involved in cardiometabolic functions. These findings suggest that maternal cadmium exposure may alter gene regulation and potentially influence cardiac development [52]. Cadmium increases ROS levels and destruction of mitochondria, as well as DNA damage in embryos [53, 54].

Mercury (Hg) exposure may occur through coal combustion, mining activities, consumption of contaminated fish, dental amalgams, and the use of mercury-containing thermometers. Various forms of Hg cross the placenta through active transport, causing deleterious effects on pregnancy through deregulation of hormonal secretion, transfer of amino acids, utilization of oxygen, and membrane flexibility [55]. Numerous studies have indicated Hg-induced oxidative stress in the placental tissue by inducing the free radical or inhibiting anti-oxidative enzyme processes, including GSH in membrane cells, which may be the cause of small gestational age [56].

Arsenic (As) exposure typically occurs through drinking water. As is identified as a human carcinogenic metal that exists in two forms, namely inorganic As and methylated organic As (inorganic As is more toxic than methylated organic As). Several studies have shown that As accumulation in placental tissue can induce the formation of free radicals, which subsequently damage placental cells through the activation of oxidative stress–related signaling pathways and may contribute to preterm delivery [57].

### Bisphenol A and its alternatives

Bisphenol A (BPA) is a synthetic chemical commonly used in the manufacture of plastics. It is present in a variety of consumer products, including polycarbonate containers, kitchenware, and epoxy resins used as coatings for metal cans, eyeglasses, and children’s toys. Used by manufacturers for over seven decades, BPA is notorious for its endocrine-disrupting effects and its tendency to migrate into food [58–60]. This migration is enhanced by environmental factors such as heat, microwave exposure, UV radiation, and repeated use. In animal studies, BPA has been linked to a wide range of adverse health effects, including metabolic problems, cardiovascular issues, liver dysfunction, nervous system disturbances, reproductive problems, and developmental abnormalities [61–65]. The mechanisms behind these effects are complex and vary depending on the type of cell or tissue involved. BPA interacts with estrogen and other hormone receptors, disrupting hormone production and affecting epigenetic processes [66–68]. Research on BPA analogs shows that many of them have similar, if not stronger, biological effects.

The effects of BPA exposure on reproductive performance are linked to changes in genes associated with implantation, such as Hoxa10, ITGB3, and EMX-2. Under normal conditions, the sex hormones estradiol and progesterone, along with their respective receptors PR and ERα, stimulate the expression of Hoxa10 in the subepithelial uterine stroma, which then suppresses the expression of EMX-2. In addition, Hoxa10, acting downstream of these sex steroids, promotes the expression of ITGB3 in endometrial epithelial cells, suggesting that these cells undergo terminal differentiation in preparation for embryo implantation [69].

There is a critical window of receptivity for blastocyst implantation [70], which is particularly narrow and highly sensitive to fluctuations in steroid hormone levels in mice [71]. Even small increases in estradiol can disrupt uterine PR (progesterone receptor) expression and gene regulation, causing the uterus to enter a refractory state. Several studies have shown that BPA can impair the development of mouse embryos, but this effect can be alleviated by tamoxifen, a selective estrogen receptor modulator [72, 73]. Therefore, the adverse effects of BPA on embryo development may be counteracted by modulating the estrogen receptor pathways. Preimplantation exposure to BPA can alter the expression of ERα (estrogen receptor α) and PR (progesterone receptor) in the mouse uterus [74]. This suggests that BPA may disrupt the coordinated actions of progesterone and estrogen, potentially impairing uterine receptivity and affecting embryo migration.

In addition, BPA triggers various biological effects by activating intracellular signaling pathways and regulating gene expression through both cell surface and nuclear receptors. Membrane receptors can activate the SRC and ERK pathways as well as the PI3K/AKT pathway, affecting cell proliferation and migration. Also, BPA can alter enzyme activity, including that of the CYP450 family and antioxidant enzymes. For example, aromatase catalyzes the conversion of androgens to estrogens, affecting endocrine function. BPA also affects the antioxidant system, leading to the accumulation of reactive oxygen species (ROS) and lipid peroxidation (LPO), which can affect cell function and cause mitochondrial dysfunction. BPA also contributes to genetic toxicity such as DNA strand breaks, oxidative DNA damage, and chromosomal mutations. Epigenetic modifications also play an important role in BPA-induced gene expression, even in the absence of DNA sequence changes [75].

Several experimental studies provide evidence that exposure to bisphenol A (BPA), an endocrine-disrupting chemical, alters the expression of molecules involved in decidualization. Both low and high doses of BPA disrupt this process by dysregulating estrogen (ER) and progesterone (PR) receptors. Low-dose BPA exposure results in decreased activity and levels of antioxidant enzymes (such as superoxide dismutase [SOD], glutathione peroxidase [GPx], heme oxygenase [HO], and catalase [CAT]), along with increased endothelial nitric oxide synthase (eNOS) activity and increased nitric oxide (NO) production through upregulation of ER and PR [76]. This imbalance induces oxidative stress and impairs decidualization. In contrast, high BPA exposure decreases ER and PR expression and disrupts decidualization through two distinct mechanisms. One pathway involves the increased expression of early growth response-1 (EGR1) through the enhanced phosphorylation of extracellular signal-regulated protein kinases 1 and 2 (ERK1/2). The other pathway involves a reduction in serum glucocorticoid–induced kinase-1 (SGK1), leading to downregulation of epithelial sodium channel-α (ENACα) and induction of oxidative stress through decreased levels and activity of antioxidant enzymes.

Then, there is mounting evidence that combined exposure to BPA from dietary and non-dietary sources during pregnancy may contribute to abnormal fetal growth. During pregnancy, BPA can pass through the placenta [77]. The fetus is theoretically more vulnerable because of absent or reduced UDP-glucoronyltransferase, which metabolize BPA to BPA-glucuronide (BPA-G) in the liver, especially during the first two trimesters [78]. Interestingly, it has been proposed that epigenetic processes may contribute to the association between prenatal exposure to bisphenol A (BPA) and childhood obesity, although human data are limited. Previous findings suggested that BPA exposure in utero may lead to altered methylation of the IGF2R gene at age 2 years. Such evidence points to a potential critical window for DNA methylation early in development that could influence BMI trajectories throughout childhood, potentially in a sex-specific manner [79].

### Bisphenol S and F

Maternal exposure to BPS or BPF can cause these chemicals to accumulate in the fetus, resulting in prolonged exposure that may interfere with normal growth and development [80]. In summary, existing research suggests that exposure to these two bisphenol analogues during pregnancy and lactation may lead to a range of problems in the offspring, including reduced fetal growth, neurological impairment, and metabolic disorders, which may persist into childhood [81–83]. In addition, exposure to BPS and BPF may be associated with an increased risk of preterm birth [84]. They are known to affect several receptor-mediated signaling pathways, including estrogen receptors α (ERα) and β (ERβ), membrane estrogen receptors (mERs), the G protein–coupled estrogen receptor (GPER), thyroid hormone receptors (TRs), androgen receptors (ARs), glucocorticoid receptors (GRs), estrogen-related receptors (ERRs), and other nuclear receptors. In addition, brain aromatase is a target of toxicity. BPS/F has also been shown to cause oxidative stress by generating reactive oxygen species (ROS). They can also affect gene transcription and disrupt post-translational protein modifications. Hormone-responsive elements (HREs) are also involved in these processes [85].

## Air pollution and embryo-fetal development

Epidemiological investigations have identified ambient air pollution, encompassing fine inhalable particulate matter (PM2.5), coarse particulate matter (PM10), carbon monoxide (CO), nitrogen dioxide (NO_2_), ozone (O_3_), and sulfur dioxide (SO_2_), as potential risk factors for infertility and adverse pregnancy outcomes [86].

Embryonic development is a highly sensitive process influenced by various environmental factors, including air pollution. Increasing evidence suggests that exposure to airborne pollutants, particularly fine PM, can disrupt early embryonic development by altering cellular differentiation and lineage specification, ultimately affecting reproductive outcomes.

A murine model study examined the effects of PM2.5 exposure on the preimplantation stage of embryonic development [87]. Mice were exposed to PM2.5, primarily derived from urban traffic emissions, both pre- and postnatally. Observations focused on embryonic development, differentiation, and the specification of cell lineages at the blastocyst stage. The results revealed that exposure to fine particulate matter significantly altered the distribution of cells between the inner cell mass (ICM) and the trophectoderm (TE), reducing the number of cells in the ICM while maintaining the total cell count in blastocysts. This suggests a deviation in cellular segregation favoring the TE lineage, which may contribute to adverse reproductive outcomes. Consistent with these findings, another study using a female murine model investigated the effects of short-term exposure to diesel exhaust particles (DEP) on fertilization and early embryonic development [88]. While ovarian response to superovulation remained unaffected, DEP exposure altered ICM morphology in blastocysts. The number of ICM cells and the ICM/TE ratio were significantly lower in DEP-exposed groups compared to controls, indicating a disruption in cell lineage segregation without altering the total cell count in blastocysts. These findings suggest that short-term DEP exposure may impair cell lineage specification within the blastocyst, potentially compromising embryo viability and development.

In human studies, air pollution has also been associated with adverse reproductive outcomes. A retrospective study involving 400 patients undergoing their first cycle of medically assisted reproduction (MAR) assessed the effects of mean PM10 exposure during the 14-day period following the last menstrual period [89]. The study found that elevated PM10 levels during the follicular phase did not negatively impact key clinical and laboratory parameters, including oocyte yield, fertilization rate, embryo morphology, embryo quality, or implantation rates. However, exposure to higher PM10 concentrations was associated with an increased risk of early pregnancy loss. This risk appeared to rise with PM levels exceeding established threshold values, suggesting that particulate matter may compromise embryonic viability rather than initial embryo quality. The authors hypothesized that experimental murine studies demonstrated that pollutants such as PM10 and DEP can disrupt ICM morphological integrity and critical factors for blastocyst growth and viability may provide a mechanistic explanation for these findings. Specifically, defects in cell lineage specification and loss of ICM integrity may represent pathways through which PM10 exposure increases the risk of early pregnancy loss. Notably, the study distinguished between biochemical pregnancies and miscarriages, revealing that the majority of adverse outcomes (73%) occurred after the detection of fetal cardiac activity. This suggests that embryo viability, rather than implantation potential, is more likely to be compromised by preconceptional exposure to elevated ambient PM10 levels. Overall, these findings indicate that exposure to air pollutants such as PM2.5, DEP, and PM10 can negatively impact embryonic development by disrupting cell lineage specification, altering blastocyst integrity, and increasing the risk of early pregnancy loss.

The evidence linking air pollution to disrupted embryonic development and early pregnancy loss raises an important question: do these adverse effects originate solely at the level of implantation and early embryogenesis, or do they stem from earlier disruptions in gametogenesis? Observational and epidemiological studies have consistently demonstrated a correlation between air pollution and adverse reproductive outcomes. However, the precise biological mechanisms underlying these associations remain largely undefined. It is plausible that air pollution negatively impacts live birth rates not only by impairing early pregnancy processes but also by exerting direct toxic effects on gametogenesis, thereby predisposing embryos to developmental failure even before implantation occurs. Given the increasing number of cryopreserved embryos in MAR, frozen embryo transfers (FETs) provide a unique opportunity to investigate this question by allowing the independent assessment of environmental influences on gamete development and early pregnancy. A recent retrospective cohort study analyzed 3657 FET cycles to examine the relationship between ambient air pollution exposure and clinical reproductive outcomes [90]. To quantify pollution exposure, air pollutant concentrations, including PM2.5, PM10, nitrogen oxides (NO, NO_2_), sulfur dioxide (SO_2_), ozone (O_3_), and carbon monoxide (CO), were monitored through a government-regulated air quality station. Pollutant levels were assessed over multiple exposure windows (24 h, 2 weeks, 4 weeks, and 3 months) prior to oocyte retrieval and embryo transfer. The study identified a significant linear association between higher exposure to PM2.5 in the 3 months preceding oocyte retrieval and a reduced likelihood of live birth following FET, independent of air quality at the time of embryo transfer. Specifically, when PM2.5 concentrations were in the highest quartile, the odds of Live birth were reduced by 34% compared to the lowest quartile. A similar trend was observed for PM10 exposure during the two weeks before oocyte retrieval, where higher concentrations were associated with a 38% decrease in live birth rates [90]. These findings strongly suggest that pollution exposure during the period of oocyte development and maturation has a lasting negative impact on reproductive success, even when embryos are later transferred under optimal conditions. This suggests that the primary mechanism by which air pollution affects IVF outcomes may be through its impact on oocyte quality rather than implantation or early pregnancy failure.

Further evidence of the detrimental impact of air pollution on embryological outcomes comes from a study investigating the effects of acute exposure to wildfire smoke on IVF outcomes. A retrospective cohort analysis conducted at a university-based fertility clinic during the 2020 Oregon wildfires examined whether a 10-day period of unhealthy air quality influenced embryo development and viability [91]. The study found that 2 days of exposure to wildfire-related pollution during in vitro fertilization and/or embryogenesis was associated with a lower number of blastocysts obtained and a higher proportion of cycles with no blastocysts available for transfer. These findings indicate that even short-term exposure to severe air pollution can negatively impact embryo viability, potentially reducing the success of ART. Notably, the study also highlights that compromised air quality inside IVF laboratories, even with air filtration systems in place, may introduce pollutants that disrupt embryonic development.

The mechanisms through which particulate pollution may affect oocyte development as well as embryo development remain to be elucidated; however, there are several physiologically plausible mechanisms. Effects may also vary depending on the type of pollution, with proposed mechanisms including the generation of oxidative stress, DNA damage, epigenetic DNA modifications, and endocrine-disrupting actions, including estrogenic, antiestrogenic, and antiandrogenic activity [92]. Given these potential pathways, understanding the extent to which air pollution interferes with reproductive and fetal development requires further investigation.

Recent literature has further explored the biological mechanisms by which ambient air pollution (particularly PM, NO_2_, and O_3_) may adversely affect birth outcomes, including suboptimal fetal growth, preterm birth, and fetal mortality [93]. Studies indicated that air pollution can impair placental function through oxidative stress, inflammation, epigenetic alterations, and endocrine disruption, ultimately compromising maternal–fetal circulation. These disruptions may contribute to impaired fetal growth and an increased risk of pregnancy complications, including preterm birth. The evidence linking air pollution to placental dysfunction underscores the broader impact of environmental pollutants on reproductive health, reinforcing the urgent need for targeted research to elucidate the mechanisms driving these adverse outcomes.

## Heat and embryo-fetal development

In mammals, global warming causes an increase in body temperature above the physiological homeothermic point (hyperthermia) with consequent organic suffering (heat stress [HS]) that leads to impaired physiological and reproductive activities [94]. Specifically, the injuries caused by heat HS on reproductive function involve both male and female components, fertilization mechanisms, as well as the early and late stages of embryo‐fetal development [95].

HS can impair embryonic development either directly or by damaging gametes at different stages of gametogenesis. In mice, exposure of testes to 42 °C for 20 min resulted in embryos approximately 20% smaller at day 10.5 compared to controls, though compensatory growth was observed later in pregnancy.

However, mating and pregnancy rates were not affected [96]. In dairy cattle, HS on day 1 post-insemination significantly reduced embryo viability, but embryos developed resistance to Maternal HS effects by day 3 [97]. Similarly, bovine embryos cultured in vitro exhibited high sensitivity to HS at early developmental stages, with repeated exposure significantly reducing blastocyst formation [98]. Later-stage embryos, such as morulae and blastocysts, develop thermotolerance, increasing resistance to HS. This adaptation is attributed to (i) antioxidant accumulation in response to heat-induced reactive oxygen species (ROS) production, (ii) HSP70 synthesis, an anti-apoptotic heat shock protein, which occurs as early as the two-cell stage in bovine and mouse embryos, and (iii) metabolic adaptation, where the embryo shifts to an energy-efficient metabolism, reducing ROS production and increasing glutathione levels [94].

Concerning fetal development, the mammalian fetus has a limited capacity for thermoregulation and is entirely dependent on maternal thermoregulatory mechanisms to maintain an optimal intrauterine environment. Under conditions of elevated maternal temperature, fetal exposure to HS can lead to increased embryonic and fetal abnormalities, including reabsorption, spontaneous abortion, or teratogenesis. Unlike prolonged hyperthermia, short-term increases in maternal temperature induced by brief radiofrequency exposures have not been associated with significant fetal malformations [66]. In humans, even mild maternal thermal stress may theoretically alter placental and umbilical blood flow due to an adaptive increase in peripheral vasodilation, which serves as a heat dissipation mechanism. This redistribution of blood volume may reduce placental heat exchange efficiency, potentially affecting fetal development [99]. Recent evidence from a comprehensive systematic review and meta-analysis, including 198 studies worldwide, demonstrated that heat exposure during pregnancy significantly increases the risk of several adverse outcomes. Specifically, heat exposure during pregnancy was associated with adverse fetal and Perinatal outcomes, particularly stillbirths, where harmful associations were found in over 10 million stillbirths [100]. These findings underscore that escalating heat exposure represents a major threat to maternal and neonatal health, with critical susceptibility windows during both early and late pregnancy.

## Summary

**Fig. 1:**
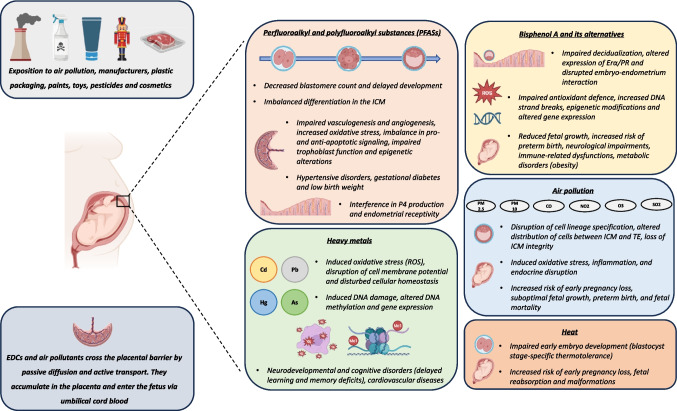
Summary of the potential effects of warming, endocrine disruptors, and air pollution on embryo development

Growing scientific evidence underscores the profound impact of environmental pollutants on embryo-fetal development, as summarized in Fig. 1. EDCs, PFASs, heavy metals air pollutants, and heat stress can disrupt cellular homeostasis and embryonic development through oxidative stress, inflammation, endocrine interference, and epigenetic modifications (Table 1). Bisphenol A and its analogues alter ERα/PR signaling, compromise decidualization, and disrupt the embryo–endometrium dialogue, contributing to impaired fetal growth, immune dysfunctions, and metabolic diseases. PFASs impair blastomere differentiation, vasculogenesis, and angiogenesis, and interfere with progesterone production, thereby affecting endometrial receptivity. Heavy metals such as cadmium and lead induce oxidative stress and DNA damage, disturb gene expression and methylation, and have been linked to neurodevelopmental and cardiovascular disorders. Air pollution, especially PM2.5 and PM10, is associated with altered cell lineage specification in the blastocyst, an increased risk of early pregnancy loss, and placental dysfunction. Finally, heat stress impairs embryo development at stage-specific windows and increases the risk of pregnancy complications through thermoregulatory failure and oxidative injury. These mechanisms highlight the urgent need to recognize environmental exposure as a critical determinant of reproductive success and developmental health.

**Table 1:** Evidence on environmental pollutants and embryo–fetal outcomes

Pollutant	Target/tissue	Main outcome(s)	Proposed mechanism(s)	Key studies
PFAS	Placenta, endometrium, early embryo	Menstrual irregularities, impaired implantation, low birth weight	Disruption of progesterone synthesis/signaling, oxidative stress, altered vasculogenesis/angiogenesis, epigenetic alterations	[16–23]
Heavy metals (Pb, Cd, Hg, As)	Placenta, cord blood, fetal tissues	Fetal growth restriction, preterm birth, neurodevelopmental delay, adverse cardiometabolic outcomes	Oxidative stress, DNA damage, disrupted methylation, interference with hormone activity	[24–57]
BPA/S/F	Placenta, endometrium, fetal tissues	Impaired implantation, fetal growth restriction, neurodevelopmental delay, metabolic disorders	Interference with hormone activity, oxidative stress, epigenetic modifications, and altered gene expression	[58–85]
Air pollution (PM2.5, PM10, NO_2_, O_3_, wildfire smoke)	Blastocyst (ICM/TE), placenta	Reduced live birth rates, early pregnancy loss, impaired blastocyst yield, fetal growth restriction	Oxidative stress, endocrine disruption, altered cell lineage specification, placental inflammation	[86–93]
Heat stress	Gametes, early embryo, fetal tissues	Reduced embryo viability, miscarriage, impaired fetal development	Hyperthermia-induced oxidative stress, impaired thermoregulation, metabolic imbalance	[94–100]

## Conclusions

Despite the overwhelming evidence that environmental pollutants pose a significant threat to reproductive health, scientific funding and policy responses remain inadequate. While epidemiological studies highlight strong associations, causative mechanisms remain elusive, limiting our ability to develop targeted interventions. Without rigorous research, regulatory frameworks remain weak, exposing future generations to irreversible reproductive harm.

Investing in comprehensive, multidisciplinary research is not optional, but it is essential. Future studies should focus on:

i) Identifying precise molecular mechanisms of reproductive toxicity;
ii) Developing biomarkers for early detection of environmental reproductive risks;
iii) Implementing stricter regulations to minimize exposure to reproductive toxins; and
iv) Enhancing ART protocols to mitigate environmental effects on embryo quality.

Environmental pollution represents a silent crisis in reproductive health, yet we remain dangerously unprepared to address its full impact. Without urgent scientific and policy-driven action, fertility rates will continue to decline, pregnancy loss will increase, and developmental disorders will become more prevalent. Investing in reproductive environmental research is not just about preserving fertility—it is about securing the future of human health.

## Data Availability

Not applicable.
